# Oligo-Carrageenan Kappa-Induced Reducing Redox Status and Activation of TRR/TRX System Increase the Level of Indole-3-acetic Acid, Gibberellin A_3_ and *trans*-Zeatin in *Eucalyptus globulus* Trees

**DOI:** 10.3390/molecules190812690

**Published:** 2014-08-20

**Authors:** Alberto González, Rodrigo A. Contreras, Gustavo Zúñiga, Alejandra Moenne

**Affiliations:** 1 Marine Biotechnology Laboratory, Faculty of Chemistry and Biology, University of Santiago of Chile, 9170022 Santiago, Chile; E-Mail: alberto.ngf@gmail.com; 2 Plant Physiology and Biotechnology Laboratory, Faculty of Chemistry and Biology, University of Santiago of Chile, 9170022 Santiago, Chile; E-Mails: rodrigo.contrerasar@usach.cl (R.A.C.); gustavo.zuniga@usach.cl (G.Z.)

**Keywords:** ascorbate, *Eucalyptus globulus*, glutathione, growth-promoting hormones, NADPH, thioredoxins

## Abstract

*Eucalyptus globulus* trees treated with oligo-carrageenan (OC) kappa showed an increase in NADPH, ascorbate and glutathione levels and activation of the thioredoxin reductase (TRR)/thioredoxin (TRX) system which enhance photosynthesis, basal metabolism and growth. In order to analyze whether the reducing redox status and the activation of thioredoxin reductase (TRR)/thioredoxin (TRX) increased the level of growth-promoting hormones, trees were treated with water (control), with OC kappa, or with inhibitors of ascorbate synthesis, lycorine, glutathione synthesis, buthionine sulfoximine (BSO), NADPH synthesis, CHS-828, and thioredoxin reductase activity, auranofine, and with OC kappa, and cultivated for four additional months. *Eucalyptus* trees treated with OC kappa showed an increase in the levels of the auxin indole 3-acetic acid (IAA), gibberellin A3 (GA_3_) and the cytokinin *trans*-zeatin (*t*-Z) as well as a decrease in the level of the brassinosteroid *epi*-brassinolide (EB). In addition, treatment with lycorine, BSO, CHS-828 and auranofine inhibited the increase in IAA, GA_3_ and *t*-Z as well as the decrease in EB levels. Thus, the reducing redox status and the activation of TRR/TRX system induced by OC kappa increased the levels of IAA, GA_3_ and *t*-Z levels determining, at least in part, the stimulation of growth in *Eucalyptus* trees.

## 1. Introduction

The principal plant growth-promoting hormones are auxins, gibberellins, cytokinins and brassinosteroids [1,2,3,4]. The auxin indole-3-acetic acid (IAA) is synthesized from tryptophan through a two-step pathway involving the enzymes tryptophan aminotransferase that synthesizes indole-3-pyruvic acid, and the flavin-monooxygenase YUCCA that synthesizes indole-3-acetic acid using O_2_ and NADPH as co-substrates [3]. The auxin IAA regulates cell division, cell expansion, cell differentiation, lateral root formation, flowering and tropic responses. In addition, active gibberellins A1, A3 and A4 are synthesized from the terpene precursor geranylgeranyl diphosphate; the latter forms the *ent*-kaurene ring that is hydroxylated in distinct positions by cythocrome P450 monooxygenases and 2-oxoglutarate-dependent dioxygenases using ascorbate as co-substrate [1,5]. Gibberellins regulate seed germination, stem elongation, flower induction, and seed development. Moreover, the most active cytokinin is *trans*-zeatin (*t*-Z), which is synthesized from adenosine nucleotides AMP, ADP or ATP that are conjugated with the isoprenoid precursor dimethylallyl diphophate (DMAPP) and hydroxylated at the end of the isoprenoid chain by a cytochrome P450 monooxygenase transforming isopentenyl adenine into *t*-Z [6]. Cytokinins regulate cell division, germination, root and shoot growth, cambial proliferation and senescence. Furthermore, the active brassinosteroid *epi*-brassinolide (EB) is synthesized from cholesterol that is transformed into campesterol, which is then hydroxylated in several positions by cytochrome P450 monooxygenases [7,8]. EB regulates cell expansion and elongation, vasculature differentiation, pollen germination, and senescence. Thus, the four main groups of growth-promoting hormones have redundant effects since they regulate overlapping processes [1,2,3,4].

The effects of growth-promoting hormones are mainly determined by the ratio among particular hormones showing positive or negative interactions. It has been shown that IAA and gibberellins display a reciprocal positive interaction since IAA regulate gibberellin metabolism [9], and gibberellins regulate the abundance of auxin efflux transporters PIN in *Arabidopsis* [10]. In addition, IAA/gibberellins displayed mostly a reciprocal negative interaction with cytokinins [2] since *t*-Z regulates auxin metabolism in *Arabidopsis* [11], and GA_3_ regulates *t*-Z levels in tomato [12]. In addition, it has been shown that auxins and gibberellins have a synergistic effect with EB, which can be negative or positive [13].

In plants, redox status is determined by the level of reducing compounds, mainly NADPH, NADH, ascorbate (ASC) and glutathione (GSH) [14]. Regarding the relationship among redox status and growth-promoting hormone levels, it has been recently observed that a triple mutant of *Arabidopsis thaliana* impaired in GSH synthesis and in cytosolic thioredoxin reductase (TRR) activities, showed normal development until the rosette stage, but failed to generate lateral organs from the inflorescence meristem producing almost naked stems as well as loss of apical dominance, vascular defects and reduced formation of lateral roots [15]. The latter characteristics are reminiscent of mutants affected in auxin transport or biosynthesis. Indeed, the triple mutant showed lower amounts of auxin, and an impaired polar transport due to a decrease in transcripts of auxin transporters [15]. The defects in organogenesis and root development were reverted by exogenous auxin or GSH application [15]. Thus, a reduction in GSH level and TRR activity induced a decrease in auxin levels and transport. This is the first direct evidence of that redox status, in particular GSH, regulates growth-promoting hormone levels. The involvement of NAD(P)H, ASC and TRR/TRX on growth-promoting hormone levels has not been analyzed until now.

Oligo-carrageenan (OC) kappa was obtained by acid hydrolysis of pure kappa carrageenan and is constituted by around 20 galactose units sulphated at C4 linked to an anhydrogalactose [16]. In previous work, we demonstrated that treatment of *Eucalyptus globulus* trees with OC kappa induced a reducing redox status due to the increase in NADPH, ASC and GSH synthesis and the increase in NADPH activates TRR/TRX system which, in turn, activates photosynthesis, C, N and S assimilation, basal metabolism and growth [17]. In addition, we showed that treatment with OC kappa activates secondary metabolism leading to an increase in terpenoid synthesis and a reprogramming of terpenoid metabolism [18].

In this work, we analyzed whether the reducing redox status and the activation of TRR/TRX system determine an increase in the level of growth-promoting hormones IAA, gibberellins A1, A3 and A4, the cytokinin *t*-Z and the brassinosteroid EB, explaining, at least in part, the increase in growth induced by OC kappa in *Eucalyptus* trees. To this end, *Eucalyptus* trees were sprayed on leaves with water (control), with OC kappa at 1 mg∙mL^−1^ or with lycorine, an inhibitor of ASC synthesis, buthionine sulfoximine (BSO), an inhibitor of GSH synthesis, CHS-828, an inhibitor of NAD(P)H synthesis, and auranofine, an inhibitor of TRR activity, and with OC kappa at 1 mg∙mL^−1^. Under these treatments, trees were grown for four additional months and the levels of IAA, GA1, GA3, GA4, *t*-Z and EB were determined.

## 2. Results and Discussion

**Figure 1:**
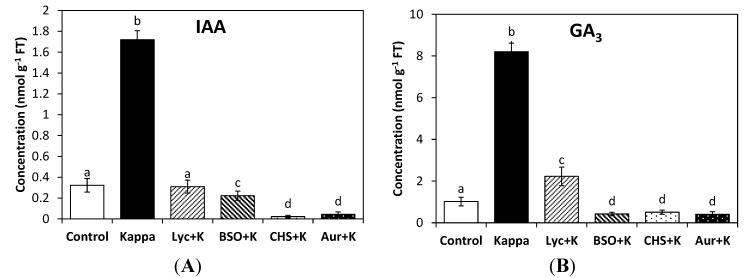
Level of indole 3-acetic acid (IAA, **A**) and gibberellin A_3_ (GA_3_, **B**), in control *Eucalyptus* trees (control) and in trees treated with OC kappa (kappa) or with lycorine and OC kappa (lyc + kappa), buthionine sulfoxime (BSO + kappa), CHS-828 and OC kappa (CHS + kappa) and auranofine and OC kappa (aur + kappa) cultivated for 4 additional months without additional treatment. The level of IAA and GA_3_ is expressed in nanomoles per gram of fresh tissue (FT). Bars indicate mean values of three independent experiments and different letters significant differences (*p* < 0.05).

The level of IAA in control *Eucalyptus* trees was 0.3 nmoles∙g^−1^ of fresh tissue (FT) and in treated trees it was 1.7 nmoles∙g^−1^ of FT, which represents a 5.6-fold increase. The increase in IAA was completely inhibited by lycorine, BSO, CHS-828 and auranofine (Figure 1A). In addition, the level of GA_3_ in control trees was 1 nmoles∙g^−1^ of FT, and in treated trees it was 8.2 nmoles∙g^−1^ of FT, which represents a 8.2-fold increase. GA_1_ and GA_4_ were not detected in control or treated trees. In addition, the increase in GA_3_ was partially inhibited by lycorine and completely inhibited by BSO, CHS-828 and auranofine (Figure 1B). Thus, reducing redox status and the activation of TRR/TRX system induced by OC kappa determine the increase in IAA and GA_3_ levels in *E. globulus* trees.

**Figure 2:**
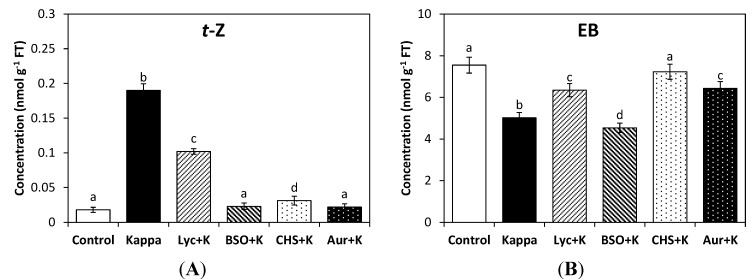
Level of *trans*-zeatin (*t*-Z, **A**) and epibrassinolide (EB, **B**) in control *Eucalyptus* trees (control), in trees treated with OC kappa (kappa) and in trees treated with lycorine and OC kappa (lyc + kappa), buthionine sulfoxime (BSO + kappa), CHS-828 and OC kappa (CHS + kappa) and auranofine and OC kappa (aur + kappa) cultivated for 4 additional months without additional treatment. The level of *t*-Z and EB is expressed in nanomoles per gram of fresh tissue (FT). Bars indicate mean values of three independent experiments and different letters significant differences (*p* < 0.05).

The level of *t*-Z in control *Eucalyptus* trees was 0.02 nmoles∙g^−1^ of FT and in treated trees it was increased 10-fold to 0.2 nmoles∙g^−1^ of FT. The increase in *t*-Z was partially inhibited by lycorine but completely inhibited by BSO, CHS-828 and auranofine (Figure 2A). The level of isopentenyl adenine (iP) slightly decreased and the level of dihydrozeatin (dZ) did not change in trees treated with OC kappa (data not shown). In addition, the level of the brassinosteroid EB in control trees was 7.5 nmoles∙g^−1^ of FT, and in treated trees it was 5 nmoles∙g^−1^ of FT, which represents a 33% decrease (Figure 2B). The level EB increased with lycorine, CHS-828 and auranofine, but not with BSO. Thus, the reducing redox status due to the increase in ASC, GSH and NADPH and the activation of TRR/TRX system induced an increase in cytkonin *t*-Z but a decrease in brassinosteroid EB levels. Therefore, OC kappa induced a concomitant increase of growth-promoting hormones IAA, GA_3_ and *t*-Z which may determine, at least in part, the stimulation of growth in *E. globulus* trees.

In this work, we demonstrated that *Eucalyptus* trees treated with OC kappa displayed a concomitant increase in IAA and GA_3_ levels, which is in accord with previous observations showing a reciprocal and positive interaction among auxin and gibberellin in other plants [9,10]. In addition, treated trees displayed an increase in *t*-Z level, which contrasts with observations showing a negative interaction among IAA/GA and *t*-Z in other plants [11,12]. In contrast, treated trees evidenced a decrease in EB levels, indicating that there is a negative interaction among IAA/GA_3_ and EB which has been previously described for gibberellins and EB in other plants [13]. Thus, OC kappa induced an increase in three main growth-promoting hormones IAA, GA_3_ and *t*-Z, but a decrease in EB.

In addition, our results indicate that the reducing redox status due to the syntheses of NADPH, ASC and GSH and the activation of TRR/TRX system induced by OC kappa, increased IAA, GA_3_ and *t*-Z levels. In this sense, it is well known that syntheses of auxin, gibberellin, cytokinin require several hydroxylations involving enzymes such as cytochrome P450 monooxygenases and flavin-dependent monooxygenases, that uses O_2_ and NADPH as co-substrates, and/or 2-oxoglutatarate-dependent dioxygenases, that uses 2-oxoglutarate and ASC as co-substrates [3,5,6,8]. In addition, it has been shown that GSH regulates the level of IAA and transport in *A. thaliana* [15]. Thus, it is not surprising that the inhibition of NADPH, ASC or GSH syntheses may inhibit the increase in growth-promoting hormone levels. In addition, we showed the increase in IAA, GA_3_ and *t*-Z levels was dependent on TRR/TRX activities, indicating that TRR/TRX system is also involved in the regulation of auxin, gibberellin and cytokinin levels, interaction that has not been reported until now. In contrast, the decrease in EB level may respond to the increase in IAA, GA_3_ and/or *t*-Z levels, and not to reducing redox status or TRR/TRX activity.

## 3. Experimental

### 3.1. Preparation of OC kappa

Twenty grams of pure (free of proteins and secondary metabolites) commercial kappa2 carrageenan (Gelymar S.A., Santiago, Chile) were solubilized in 2 L of water at 60 °C. Concentrated HCl (36.2 N) was added to reach a final concentration of 0.1 N, the solution was incubated for 45 min at 60° and then NaOH 1 M was added to obtain pH 7. A sample of 10 µL of the depolymerized carrageenan corresponding to oligo-carrageenan (OC) kappa was analyzed by electrophoresis in an agarose gel (1.5% w/v) using 100 V for 1 h and dextran sulphate of 8 and 10 kDa as standards (Sigma, St. Louis, MO, USA). The gel was stained with 15% w/v Alcian blue dye in 30% v/v acetic acid/water for 1 h at room temperature and washed with 50% v/v acetic acid/water for 1 h. OC kappa was visualized as a relative discrete band of around 10 kDa.

### 3.2. Plant Culture, Treatment with OC Kappa, with Inhibitors and OC Kappa, and Measurement of Height

*E. globulus* trees with an initial height of 30 cm (n = 10 for each group) were cultivated outdoors in plastic bags containing compost. *E. globulus* trees were sprayed in the upper and lower part of leaves with 5 mL per plant of water/methanol 9:1 v/v (control group, n = 10), an aqueous solution of OC kappa at a concentration of 1 mg∙mL^−1^ (treated group 1, n = 10), a water/methanol solution of 250 μM CHS-828, an inhibitor of nicotinamide phosphoribosyl transferase [19] and of NAD(P)H synthesis (treated group 2, n = 10), a water/methanol solution of 250 μM lycorine, an inhibitor of galactonolactone dehydrogenase [20] and of ASC synthesis (treated group 3, n = 10), a water/methanol solution of 1.5 mM buthionine sulfoximine (BSO), an inhibitor of γ-glutamylcysteine synthase [21] and of GSH synthesis (treated group 4, n = 10), and with auranofin, an inhibitor of TRR activity [22], and with OC kappa at a concentration of 1 mg∙mL^−1^. Trees of treated groups 2, 3 and 4 were treated twice with CHS-828, auranofine, lycorine or BSO and after two weeks they were treated with OC kappa once a week, four times in total, and cultivated without any additional treatment for four months. Leaves were obtained from the middle part of control and treated trees, and pooled into three groups to perform further analysis (n = 3). The height of trees was determined using a measuring tape.

It is important to mention that different concentration CHS-828, lycorine, BSO and auranofin were sprayed on *Eucalyptus* leaves to determine the optimal concentration of each inhibitor (data not shown). In addition, it was determined that the optimal concentration of CHS-828 decreased NADPH content, the optimal concentration of lycorine inhibited galatonolactone dehydrogenase (GLDH) activity, the optimal concentration of BSO inhibited γ-glutamylcysteine synthase (γ-GCS) activity, and the optimal concentration of auranofin inhibited TRR activity at four months of culture without additional treatment.

### 3.3. Extraction and Quantification of Plant Hormones

Plant hormones were extracted and analyzed as described in Pan *et al.* [23] with some modifications. *Eucalyptus* leaves (1 g of FT) were obtained from the middle part of control and treated Eucalyptus trees (n = 3 for control and treated groups). Leaves were homogenized with liquid nitrogen in a mortar and 4 mL of 30% (v/v) isopropanol-15 mM HCl were added. The mixture was shaken at 4 °C for 30 min to extract plant hormones. Two mL of dichloromethane were added; the mixture was shaken at 4 °C for 30 min and centrifuged at 14,000 rpm for 15 min, and the alcoholic solution containing hormones was recovered. The alcoholic supernatant was concentrated using a flux of nitrogen (g) to reach a final volume of 180 µL.

Plant hormones were detected and quantified using an HPLC-ESI-MS/MS system (Agilent 1200 series, MS/MS 5420, Agilent Technologies, Santa Clara, CA, USA). The mobile phase was prepared with 0.1% of formic acid (A) and 0.1% of formic acid in methanol (B). A sample of 20 µL was separated using a C18 reverse phase column (150 × 4.6 mm, 5 µm, Agilent, XDB-C18) with a flow rate of 0.3 mL min^−1^ at room temperature. The elution was done using linear steps of 0 to 2 min 30% of B, 2 to 20 min increasing to 100% of B, 20 to 22 min with 100% of B and 22 to 25 min reducing to 30% of B. The MS/MS detection was performed using a multiple monitoring reaction mode, −4500 V, 25 psi and 10 L·min^−1^ of nitrogen flow. Twenty µL of a mixture of deuterated standards for IAA, GA_1_, GA_3_, GA_4_, *t*-Z, iP, dZ and EB at a concentration of 50 ng∙mL^−1^ were added to each sample. For the detection in the negative mode, the mass-to-charge (*m/z*) ratio of IAA and GA_3_ were (174.0 → 129.6; retention time (RT) = 15.5) and (345.1 → 142.7; RT = 17.30), respectively, and in the positive mode the *m/z* ratios for iP, *t*-Z, dZ and EB were (204 → 136, RT = 6.2) (220.2 → 136.2; RT = 5.2), (220.2 → 136.2, RT = 10.6) and (482 → 204.2, RT = 3.3), respectively. For the detection of internal standard in a negative mode the *m/z* ratios for d_5_-IAA and d_2_-GA_3_ were (347.1 → 142.7) and (347.1 → 142.7), respectively, and in the positive mode the *m/z* ratios for d_6_-iP, d_5_-*t*-Z, d_5_-dZ and d_3_-EB were (210 → 142), (225.2 → 136.2), (225.2 → 136.2) and (485 → 207), respectively.

### 3.4. Statistical Analysis

Significant differences were determined by two-way analysis of variance (ANOVA) followed by Tukey’s multiple comparison tests (*T*) at 95% confidence interval (*p* < 0.05). Requirements of normality and homogeneity of variance were tested using Kolmogorov-Smirnov and Bartlett Tests, respectively [24]. Mean values were determined using three independent samples.

## 4. Conclusions

In this work, we showed that a reducing redox status due to the increase in ASC, GSH and NADPH levels and the activation of the TRR/TRX system determine the increase in growth-promoting hormones IAA, GA_3_ and *t*-Z levels explaining, at least in part, the stimulation of growth induced by OC kappa in *Eucalyptus* trees.
